# A Genetic Variant in miR-196a2 Increased Digestive System Cancer Risks: A Meta-Analysis of 15 Case-Control Studies

**DOI:** 10.1371/journal.pone.0030585

**Published:** 2012-01-24

**Authors:** Jing Guo, Mingjuan Jin, Mingwu Zhang, Kun Chen

**Affiliations:** Department of Epidemiology and Health Statistics, School of Public Health, Zhejiang University, Hangzhou, Zhejiang, China; Dartmouth College, United States of America

## Abstract

**Background:**

MicroRNAs (miRNAs) negatively regulate the gene expression and act as tumor suppressors or oncogenes in oncogenesis. The association between single nucleotide polymorphism (SNP) in miR-196a2 rs11614913 and the susceptibility of digestive system cancers was inconsistent in previous studies.

**Methodology/Principal Findings:**

An updated meta-analysis based on 15 independent case-control studies consisting of 4999 cancer patients and 7606 controls was performed to address this association. It was found that miR-196a2 polymorphism significantly elevated the risks of digestive system cancers (CT vs. TT, OR = 1.25, 95% CI = 1.07–1.45; CC vs. TT, OR = 1.38, 95% CI = 1.13–1.67; CC/CT vs. TT, OR = 1.29, 95% CI = 1.10–1.50; CC vs. CT/TT, OR = 1.14, 95% CI = 1.01–1.30; C vs. T, OR = 1.15, 95% CI = 1.05–1.26). We also found that variant in miR-196a2 increased the susceptibility of colorectal cancer (CRC) (CT vs. TT, OR = 1.23, 95% CI = 1.04–1.44; CC vs. TT, OR = 1.32, 95% CI = 1.08–1.61; CC/CT vs. TT, OR = 1.25, 95% CI = 1.07–1.46; C vs. T, OR = 1.15, 95% CI = 1.05–1.28), while the association in recessive model (CC vs. CT/TT, OR = 1.16, 95% CI = 0.98–1.38) showed a marginal significance. Additionally, significant association between miR-196a2 polymorphism and increased risk of hepatocellular cancer (HCC) was detected. By stratifying tumors on the basis of site of origin, source of controls, ethnicity and allele frequency in controls, elevated cancer risks were observed.

**Conclusion/Significance:**

Our findings suggest the significant association between miR-196a2 polymorphism and increased susceptibility of digestive system cancers, especially of CRC, HCC and Asians. Besides, C allele may contribute to increased digestive cancer risks.

## Introduction

MicroRNAs (miRNAs) are endogenous, small non-coding and have a length of 18–25 nucleotides RNAs. miRNAs can interact with messenger RNAs (mRNAs) by binding to 3′ un-translated regions (UTRs) and lead to the degradation or translational repression of mRNAs. Studies revealed that miRNAs played key roles in various biological processes including cell growth regulation, differentiation, apoptosis and tumorigenesis [1], [2], [3]. miRNAs regulate approximately 30% of human genes and exhibit a remarkable contribution to carcinogenesis [2], [4]. Aberrant modulation of specific miRNAs was considered to be a crucial event of diverse diseases including cancers [5] although the detailed process of miRNAs expression and mutation are still ambiguous. Moreover, some studies detected that miRNAs participated in the etiology, progression and prognosis of cancers, such as non-small cell lung cancer [6] and hepatocellular carcinoma [7]. Several possible mechanisms, including genetic and epigenetic alternations, have been proposed. SNPs in miRNAs are marked as novel genetic variations which may modify the cancer susceptibilities [8]. Genetic variant in miR-196a2 had been demonstrated to be associated with some cancer risks, but different studies showed conflicting associations. Meta-analysis on breast cancer, lung cancer and other cancers revealed that rs11614913 was a functional SNP and had potential ability to modify the cancer risks [9], [10], [11], [12], [13].

As we know, the above-referenced meta-analysis included gastric cancer (GC), HCC and other digestive cancers for the SNP in miR-196a2. However, by the limitation of inadequate publications, they did not calculate pooled ORs of digestive system cancers comprehensively. To improve the efficiency of meta-analysis on digestive cancers and reduce the potential between-study heterogeneity which might derive from various cancers in diverse systems, we focused on digestive system cancers only and added more recent publications on CRC [14], [15], [16] and HCC [17] in this study. We also contacted the authors to request for genotype frequencies about oral cavity squamous cancer (OSCC) and pharynx squamous cancer (PSCC) [18], [19] which were not shown in published articles. In addition, an unpublished case-control study on CRC which was performed by Mingwu Zhang et al at the Molecular Epidemiology Laboratory in Zhejiang University School of Medicine was collected. Overall, 9 datasets from 7 studies (including 2875 cases with digestive cancers and 5556 controls) which had not been studied in previous meta-analysis were additionally included in our study. And we performed this meta-analysis focusing on the following issues: (a) What is the association between miR-196a2 polymorphism and the susceptibility of digestive system cancers, especially of colorectal cancer? (b) Would changes in tumor sites, demographic characteristics and other factors transform this association significantly?

## Materials and Methods

### Identification of eligible studies

**Figure 1:**
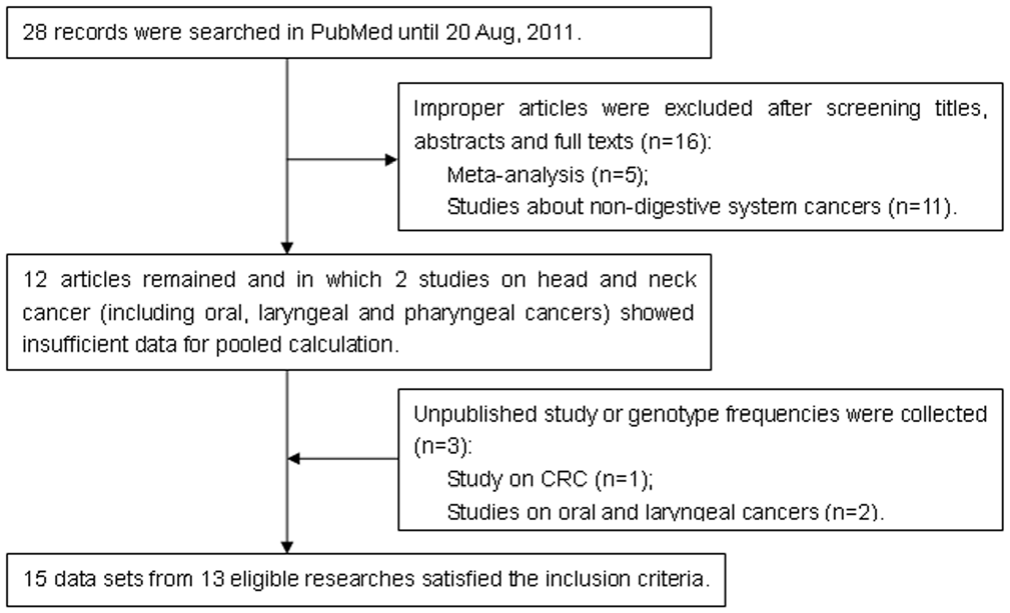
Flow diagram of studies identification.

A systematic search in PubMed was conducted using a retrieving query formulation “(microrna 196a2 OR rs11614913) polymorphisms cancer” (last search updated on 20 Aug, 2011). We also searched references in published articles and reviews on this topic in PubMed. Eligible studies were selected according to the following explicit inclusion criteria: (a) Study was designed using the methodology of a case-control study. (b) The association between miR-196a2 polymorphism and digestive system cancer risks was explored. (c) There was sufficient data for the computation of odds ratios and corresponding 95% confidence intervals (ORs, 95% CIs). (d) Cases with carcinomas were diagnosed by histopathology. Moreover, we also contacted some researchers to request unpublished study outcomes and detailed datasets for pooled calculation (Figure 1).

### Data extraction

Two investigators (Guo and Jin) screened titles, abstracts and full texts independently using a standardized screening guide. Data extraction was carried out independently after the concealment of authors, journals, supporting organizations and funds to avoid investigators' bias. After data abstraction, discrepancies and differences were resolved by consensus and discussion.

Characteristics of enrolled studies were assigned to the structured form (Table 1), including first author's name, publication time, study country origin, ethnicity, cancer type, source of controls, genotyping method, matched criteria between cases and controls, sample size, C allele frequency in controls (Table S1), genotype frequency distribution and quality scores.

**Table 1:** Characteristics of eligible studies in meta-analysis.

First author	Publication	Country	Ethnicity	Control	Cancer	Genotyping	Matching criteria	Quality	HWE
	Year	origin		source	type	method		score	
Zhan	2011	China	Asian	HB	CRC	PCR-RFLP	age/sex	6.5	Y
Chen	2010	China	Asian	HB	CRC	PCR-LDR	age/sex	7	Y
Zhu	2011	China	Asian	HB	CRC	TaqManSNP	age/sex	7.5	Y
Zhang	2011 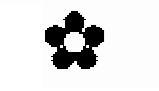	China	Asian	PB	CRC	PCR-RFLP	age/sex	7.5	N
Wang	2010	China	Asian	PB	ESCC	SNaPshot	age/sex/area	7	Y
Srivastava	2010	Indian	Caucasian	PB	GBC	PCR-RFLP	age/sex	6.5	Y
Okubo	2010	Japan	Asian	HB	GC	PCR-RFLP	UK	6.5	Y
Peng	2010	China	Asian	HB	GC	PCR-RFLP	age/sex	6.5	Y
Li	2010	China	Asian	HB	HCC	PCR-RFLP	UK	6	Y
Qi♣	2010	China	Asian	HB	HCC	PCR-LDR	UK	6.5	Y
Akkiz	2011	Turkey	Caucasian	HB	HCC	PCR-RFLP	age/sex/smoke	7	Y
Christensen	2010	USA	Caucasian	PB	OSCC	TaqManSNP	age/sex/residence	8.5	Y
Liu	2010	USA	Caucasian	HB	OSCC	PCR-RFLP	age/sex	7.5	Y
Christensen	2010	USA	Caucasian	PB	PSCC	TaqManSNP	age/sex/residence	8.5	Y
Liu	2010	USA	Caucasian	HB	PSCC	PCR-RFLP	age/sex	7.5	Y

CRC: colorectal cancer; ESCC: esophageal squamous cell carcinoma; GBC: gallbladder cancer; GC: gastric cancer; HCC: hepatocellular cancer; OSCC: oral cavity squamous cancer; PSCC: pharynx squamous cancer; UK: unknown; HWE: Hardy-Weinberg equilibrium; Y: genotype frequency distribution agreed to HWE in controls; N: genotype frequency distribution disagreed to HWE in controls; PCR-RFLP: polymerase chain reaction-restriction fragment length polymorphism; PCR-LDR: polymerase chain reaction-ligation detection reaction; HB: hospital-based; PB: population-based; 
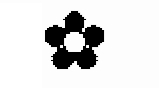
: study year; Qi♣: HBV patients without HCC were controls and HBV patients with HCC were cases in the study of Qi.

### Methodological quality assessment

Three reviewers (Guo, Jin and Zhang) independently evaluated the quality of selected studies by scoring according to a set of predetermined criteria (Table S2) which was extracted and modified from previous studies [20], [21], [22]. Quality scores ranged from 0 to 10 and the studies with higher scores presented better quality. Disagreements were resolved by discussion.

### Statistical analysis

Crude ORs and corresponding 95%CIs were calculated to investigate the association strength between miR-196a2 polymorphism and the susceptibility of digestive system cancers. Pooled ORs were obtained from combination of single studies by heterozygote comparison (CT vs. TT), homozygote comparison (CC vs. TT), dominant and recessive models (CC/CT vs. TT, CC vs. CT/TT), allelic comparison (C vs. T) respectively. We used chi-square-based Q-test [23] and the I^2^ index [24] to check the heterogeneity among different studies. When Q-test showed the existence of notable heterogeneity (P-value less than 0.10 and/or I^2^ index more than 50%,), we used the random-effects model (DerSimonian and Laird method) [25]; otherwise, the fixed-effects model (Mantel and Haenszel method) was conducted [26].

Stratification by tumor site, source of controls, ethnicity and allele frequency in controls was conducted. All cancers were categorized into two groups: digestive tract cancer and digestive gland cancer. Eligible studies were classified into population-based and hospital-based according to control source. The subjects were classified by ethnicity into Caucasian group and Asian group. We also classified the selected studies into C>T (C allele frequency more than T allele frequency) group and C≤T (C allele frequency less than or equivalent to T allele frequency) group by allele frequency in controls.

Hardy-Weinberg equilibrium (HWE) in control population was judged by the chi-square test. P-value less than 0.05 was considered to be a state of disequilibrium. Publication bias was diagnosed with Egger's linear regression method [27], [28] and funnel plot. The P-value less than 0.05 in Egger's linear regression indicated the presence of potential publication bias. The standard error of logarithm for OR was plotted against its OR in funnel plot. Begg's funnel plot was also plotted to detect the publication bias and influence of individual study on pooled OR. Log OR was plotted versus standard error of Log OR for each included study in Begg's funnel plot [29]. And asymmetric or incomplete funnel-shaped plots demonstrated publication bias also. In the one-way sensitivity analysis, we excluded one single study each time, and the new pooled results could reflect the influence of that deleted study to the overall summary OR.

The frequency distributions of C allele in Asians and Caucasians were compared using chi-square test. All statistical analysis was implemented with SAS 9.2 software (SAS Institute Inc., Cary, NC, USA), STATA 11.0 (STATA Corp, College Station, Texas) and RevMan 5.1 (http://ims.cochrane.org/revman/download). All P-values were two-sided.

## Results

### Studies characteristics

13 eligible studies including 12 published studies [14], [15], [16], [17], [18], [19], [30], [31], [32], [33], [34], [35] and 1 unpublished one were collected in this meta-analysis according to the inclusion criteria. Characteristics of these studies were presented in Table 1 and the genotype frequency distribution was shown in Table S1.

Among studies on head and neck squamous cell carcinoma (HNSCC, which included oral, pharyngeal and laryngeal cancers) [18], [19], laryngeal cancer in respiratory system was not used. We considered patients with oral cancer and pharyngeal cancer as separate groups and pooled them into quantitative analysis independently. Therefore, this meta-analysis employed 15 separate case-control studies, including 4999 cases and 7606 controls, for the polymorphism of miR-196a2.

12 studies were matched for age, sex and/or residence, smoking, alcohol consumption [14], [15], [16], [17], [18], [19], [32], [34], [35]; 9 studies collected Asians as subjects and the other 6 investigated Caucasians; C allele frequency of controls was the minor allele frequency (MAF) in 7 studies and T allele frequency was MAF in the 8 studies remained; controls in 10 studies were hospital-based and controls of the other studies were population-based; 11 studies described alimentary tract cancers and 4 studies focused on tumors in digestive glands. To dilute the potential confounding bias of HBV infection in the study of Qi et al [33], we kept the HBV patients without HCC as controls and the HBV patients with HCC as cases.

Genotypes in all studies were detected with genetic DNA from blood samples using 4 genotyping methods totally. 13 out of 15 studies checked genotypes for quality control. Genotype distribution of controls in all studies was consistent with HWE, except for Mingwu Zhang's study on CRC.

### Publication bias

**Figure 2:**
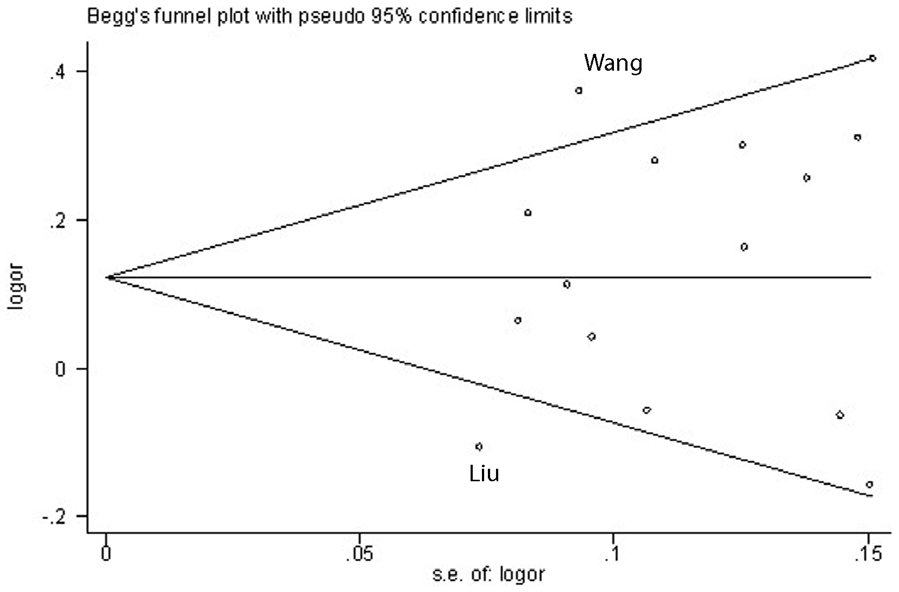
Begg's funnel plot of publication bias. Log OR is plotted versus standard error of Log OR for each included study. Every circle dot represents a separate study for the indicated association by allele contrast (C over T). Wang's study on ESCC (upper dot) and Liu's study on PSCC (lower dot) locate outside of pseudo 95% CI limits evidently.

We found no significant evidence of publication bias (P-value>0.05) in any comparison model using Egger's linear regression method. Furthermore, the shape of funnel plot for the allele contrast (C vs. T) showed approximately symmetric and inverted funnel-shaped (Figure S1). Begge's funnel plot (C vs. T) did not reveal any remarkable asymmetry in the distribution of scattered points (Figure 2). Among all studies included, Wang's study on ESCC [35] and Liu's on PSCC [19] deviated from other symmetrically distributed studies. When these two studies were deleted, I^2^ decreased from 63% (Ph = 0.0005) to 42% (Ph = 0.05). While the summary OR for allele contrast (C vs. T) still kept significant (OR = 1.15, 95%CI = 1.06–1.25), and this result was similar to pooled OR without deletion of any study (OR = 1.15, 95%CI = 1.05–1.26).

### Test of heterogeneity

Between-study heterogeneities and corresponding quantitative degrees in all comparisons and subgroups, were shown in Table S3. After stratification, the heterogeneities decreased obviously in the subgroups of CRC, GC, digestive gland, HCC, hospital-based controls, and C≤T group (Ph>0.10 and I^2^<50% in most genetic comparisons).

### Sensitivity analysis

We deleted one single study from the overall pooled analysis each time to check the influence of the removed data set to the overall ORs. Two studies (Wang (ESCC) [35] and Liu (PSCC) [19]) changed the between-study heterogeneities materially in heterozygote comparison and recessive model respectively. After the deletion of anyone of the two studies mentioned, the heterogeneity vanished, while the association still kept significant (Table S4).

### Meta-analysis results

The association strength between miR-196a2 polymorphism and the susceptibility for digestive system cancers are shown in Table 2. Overall, there was a statistically increased risk of digestive system cancers in every genetic comparison (CT vs. TT, OR = 1.25, 95% CI = 1.07–1.45; CC vs. TT, OR = 1.38, 95% CI = 1.13–1.67; CC/CT vs. TT, OR = 1.29, 95% CI = 1.10–1.50; CC vs. CT/TT, OR = 1.14, 95% CI = 1.01–1.30; C vs. T, OR = 1.15, 95% CI = 1.05–1.26).

**Table 2:** Pooled ORs and 95%CIs of stratified meta-analysis.

Stratification	N	CT vs. TT	CC vs. TT	CC/CT vs. TT	CC vs. CT/TT	C VS. T
		OR (95% CI)	OR (95% CI)	OR (95% CI)	OR (95% CI)	OR (95% CI)
Digestive cancers	15	1.25(1.07–1.45)*	1.38(1.13–1.67)*	1.29(1.10–1.50)*	1.14(1.01–1.30)*	1.15(1.05–1.26)*
Tumor site						
Alimentary tract	11	1.23(1.03–1.48)*	1.32(1.05–1.65)*	1.26(1.04–1.51)*	1.12(0.98–1.28)	1.13(1.02–1.25)*
CRC	4	1.23(1.04–1.44)*	1.32(1.08–1.61)*	1.25(1.07–1.46)*	1.16(0.98–1.38)	1.15(1.05–1.28)*
GC	2	1.07(0.85–1.34)	1.24(0.94–1.65)	1.12(0.90–1.39)	1.22(0.96–1.55)	1.12(0.98–1.28)
ESCC	1	2.42(1.66–3.55)*	2.67(1.77–4.04)*	2.51(1.74–3.62)*	1.35(1.02–1.78)*	1.45(1.21–1.75)*
OSCC	2	1.00(0.47–2.13)	1.07(0.64–1.80)	1.03(0.53–1.98)	1.05(0.87–1.29)	1.04(0.91–1.19)
PSCC	2	1.35(0.79–2.32)	1.33(0.49–3.60)	1.36(0.66–2.83)	1.00(0.59–1.68)	1.09(0.72–1.63)
Digestive gland	4	1.30(1.02–1.65)*	1.64(1.24–2.17)*	1.38(1.10–1.74)*	1.24(0.85–1.79)	1.20(0.96–1.51)
HCC	3	1.27(0.99–1.64)	1.79(1.31–2.43)*	1.41(1.11–1.79)*	1.49(1.16–1.91)*	1.32(1.14–1.54)*
GBC	1	1.50(0.72–3.12)	1.04(0.51–2.11)	1.20(0.60–2.41)	0.74(0.51–1.07)	0.85(0.64–1.15)
Source of control						
HB	10	1.21(1.08–1.36)*	1.37(1.12–1.66)*	1.24(1.12–1.38)*	1.18(1.00–1.40)	1.16(1.05–1.28)*
PB	5	1.37(0.84–2.22)	1.39(0.84, 2.30)	1.36(0.85–2.19)	1.09(0.94–1.27)	1.11(0.91–1.36)
Ethnicity						
Asian	9	1.26(1.05–1.50)*	1.47(1.18–1.82)*	1.32(1.10–1.57)*	1.25(1.11–1.40)*	1.20(1.10–1.31)*
Caucasian	6	1.26(0.93–1.72)	1.26(0.88–1.80)	1.25(0.91–1.72)	1.02(0.83–1.27)	1.07(0.91–1.26)
Allele frequency in controls						
C>T	8	1.36(1.00–1.85)	1.45(1.01–2.07)*	1.39(1.02–1.90)*	1.11(0.91–1.36)	1.15(0.98–1.34)
C≤T	7	1.18(1.04–1.34)*	1.33(1.14–1.55)*	1.22(1.08–1.37)*	1.20(1.05–1.37)*	1.15(1.07–1.24)*

N: involved studies' number; CT vs. TT: Heterozygote comparison; CC vs. TT: Homozygote comparison; CC/CT vs. TT: Dominant model; CC vs. CT/TT: Recessive model; C VS. T: Allele contrast; Random model was chosen for data pooling when P-value<0.10 and/or I^2^>50%; otherwise fixed model was used;

*: OR had statistical significance with corresponding 95%CI not including 1.

Tumor site, source of controls, ethnicity and allele frequency in controls were taken into consideration for subgroup analysis. The forest plots of dominant models (CC/CT vs. TT) in different subgroups were shown in Figure S2. Comparing with genotype TT, heterozygote CT (OR = 1.23, 95% CI = 1.03–1.48), homozygote CC (OR = 1.32, 95% CI = 1.05–1.65), combination of CT/CC (OR = 1.26, 95% CI = 1.04–1.51) predominantly increased incidences of cancers in alimentary tract. And we also found that C allele carriers had more risks of digestive tract cancers (C vs. T, OR = 1.13, 95% CI = 1.02–1.25), but no significant result was observed in recessive model (CC vs. CT/TT, OR = 1.12, 95% CI = 0.98–1.28).

Significant association between SNP rs11614913 and increased risks of digestive gland cancers was found in three genetic models (CT vs. TT, OR = 1.30, 95% CI = 1.02–1.65; CC vs. TT, OR = 1.64, 95% CI = 1.24–2.17; CC/CT vs. TT, OR = 1.38, 95% CI = 1.10–1.74), except for recessive model (CC vs. TT, OR = 1.24, 95% CI = 0.85–1.79) and allele contrast (C vs. T, OR = 1.20, 95% CI = 0.96–1.51). Additionally, we demonstrated that this locus polymorphism was significantly linked to higher risks for CRC (CT vs. TT, OR = 1.23, 95% CI = 1.04–1.44; CC vs. TT, OR = 1.32, 95% CI = 1.08–1.61; CC/CT vs. TT, OR = 1.25, 95% CI = 1.07–1.46; C vs. T, OR = 1.15, 95% CI = 1.05–1.28), but a marginal significance was found in recessive model (CC vs. CT/TT, OR = 1.16, 95% CI = 0.98–1.38). We also observed increased susceptibility of HCC in homozygote comparison (OR = 1.79, 95% CI = 1.31–2.43), dominant model (OR = 1.41, 95% CI = 1.11–1.79), recessive model (OR = 1.49, 95% CI = 1.16–1.91) and allele contrast (OR = 1.32, 95% CI = 1.14–1.54). We just found a marginal significance in homozygote comparison (OR = 1.27, 95% CI = 0.99–1.64) in HCC study. Compared with CRC and HCC, no significant associations were found in GC, OSCC and PSCC.

With consideration of control source, studies with hospital-based controls showed elevated risks in four genetic comparisons (CT vs. TT, OR = 1.21, 95% CI = 1.08–1.36; CC vs. TT, OR = 1.37, 95% CI = 1.12–1.66; CC/CT vs. TT, OR = 1.24, 95% CI = 1.12–1.38; C vs. T, OR = 1.16, 95% CI = 1.05–1.28) and an edge effect was obtained in recessive model (OR = 1.18, 95% CI = 1.00–1.40). However, studies with population-based controls presented no significant association.

For the Asian group, every genetic comparison produced significantly increased risks (CT vs. TT, OR = 1.26, 95% CI = 1.05–1.50; CC vs. TT, OR = 1.47, 95% CI = 1.18–1.82; CC/CT vs. TT, OR = 1.32, 95% CI = 1.10–1.57; CC vs. CT/TT, OR = 1.25, 95% CI = 1.11–1.40; C vs. T, OR = 1.20, 95% CI = 1.10–1.31) while no significant associations were detected in Caucasian group.

In the subgroup of C>T, remarkably elevated cancer risks were found in homozygote comparison (OR = 1.45, 95% CI = 1.01–2.07) and dominant model (OR = 1.39, 95% CI = 1.02–1.90). While we did not find significant associations among heterozygote comparison (OR = 1.36, 95% CI = 1.00–1.85), recessive model (OR = 1.11, 95% CI = 0.91–1.36) and allele contrast (OR = 1.15, 95% CI = 0.98–1.34). Meanwhile, significant association between miR-196a2 polymorphism and increased risk of digestive system cancer was also found in the C≤T group (CT vs. TT, OR = 1.18, 95% CI = 1.04–1.34; CC vs. TT, OR = 1.33, 95% CI = 1.14–1.55; CC/CT vs. TT, OR = 1.22, 95% CI = 1.08–1.37; CC vs. CT/TT, OR = 1.20, 95% CI = 1.05–1.37; C vs. T, OR = 1.15, 95% CI = 1.07–1.24).

When we compared C allele frequency in Asians with that in Caucasians, Zhang's study on CRC was excluded due to its HWE disequilibrium in controls. C allele frequency of miR-196a2 ranged from 0.419 to 0.754 across Asian and Caucasian controls. In Asian controls, C allele frequency accounted for 45.60% which was significantly lower than that in Caucasian controls (59.90%,χ^2^ = 222.32, P<0.0001 ). A former study reported a parallel observation [9].

The MOOSE Checklist for our study was shown as Table S5.

## Discussion

miRNAs participate in diverse biological processes and is regarded as a key factor in oncogenesis. SNP in miR-196a2 rs11614913 was thought to be implicated in altered expression and function of mature miRNAs, thus contributed to modified cancer risks. Many studies demonstrated variant in rs11614913 was significantly associated with the susceptibility of various cancers. Hong et al. found that carriers with TC/CC genotype of miR-196a2 had higher risks for non-small cell lung cancer (NSCLC) comparing with TT carriers [36]. Comparing with TT genotype, Hu et al. observed that CC or CC/CT genotypes significantly increased breast cancer risks [37]. Similar results were also found in glioma [38], prostatic cancer [39] and other kinds of cancers.

Further more, SNPs in miRNAs can occasionally disturb the gene or protein expression and result in pathogenicity [40]. Zhan and his colleagues reported that the expression levels of miR-196a in CC and CC/CT genotypes were higher than those in TT genotype in CRC [15]. Li et al. also found that CC and CC/CT genotypes increased the expression level of miR-196a in HCC patients with HBV infection comparing with TT genotype [30]. Hu et al. found that the expression level of miR-196a in CC genotype carriers was significantly lower than that in CT or TT carriers with NSCLC [6]. Additionally, compared with CT/TT genotype, CC genotype of miR-196a2 predominantly decreased the survival time of NSCLC patients in Hu's study [6]. Thus Hu and his colleagues proposed the genetic variant in this locus to be a prognostic biomarker for NSCLC.

Our study showed that the presence of C allele significantly increased the risk of digestive system cancers with the comparison to T allele. This finding indicates that the genetic variant in miR-196a2 may crucially modify the susceptibility of digestive system cancers. Previous meta-analysis which described cancers locating in multiple systems of organism supported our finding [9], [13].

We found that miR-196a2 polymorphism, in stratified analysis by cancer site, was statistically related with elevated cancer risks in the alimentary tract group and digestive gland group. Moreover, significantly increased risks were found in CRC and HCC. However, we did not observe any significant association between the genetic variant and the susceptibility of GC, OSCC and PSCC. There are some possibilities for this discrepancy among tumor sites. Firstly, the tissue specificity leads to different cancer susceptibilities in different tissues. Secondly, the relative small amount of eligible studies in stratified analysis might induce significant/insignificant association by chance due to insufficient statistical power [41]. Two previous meta-analysis reported insignificant association between miR-196a2 polymorphism and HCC risks [9], [13], which was inconsistent to our finding. We infer that fewer included studies and the neglect of HBV infection in controls might lead to insignificant results in previous meta-analysis.

In the subgroup of ethnicity, we found significant association between miR-196a2 polymorphism and increased risks of digestive system cancers in Asians but not in Caucasians. A former meta-analysis reported a parallel observation to us [9]. Inconsistency between the two ethnicities can be explained by the possibility that different ethnic groups live with multiple life styles and environmental factors and thus yield diverse gene-environment interactions [42]. And different populations carry different genotype and/or allele frequencies of this locus polymorphism and may lead to various degrees of cancer susceptibility [43]. Relative small sample size in Caucasians might cause the inconspicuousness also.

The majority (70%, 7/10) of studies with hospital-based controls recruited Asians as tested subjects and we found significantly increased risks in this subgroup. While most (60%, 3/5) studies with population-based controls investigated Caucasians and we did not found any significant results in this subgroup. So the mentioned ethnic interpretations are available to the inconsistency in control source stratification. And the possible selection bias in controls with different matched criteria and sample size may also be the reasons. Chu's meta-analysis study also reported significantly increased cancer risks in Asians but not in Caucasians [9].

Disagreements in the stratification of allele frequency in controls might attribute to above interpretations for ethnic effect to some extent.

Some advantages can be highlighted in our study. On one hand, this meta-analysis shed light on the association between miR-196a2 polymorphism and increased risks of digestive system cancers, CRC and HCC comprehensively and systematically. On the other hand, the inclusion of an unpublished study on CRC and the collection of unpublished genotype frequency of OSCC and PSCC strengthened the power and persuasion of our inference. Further more, all included studies had acceptable quality (scored at least 6). Limitations of this study should be noticed at the same time. Firstly, genetic factors, tumor biological characteristics and their interactions with environmental factors produce evident influences to the cancer susceptibility and tumorigenesis. Different cancers have different risk factors and diverse sensitivities to them. For instance, Heliobacter pylori infections and smoking may increase the incidence of gastric cancer. And hepatitis B, C virus infections and exposure of aflatoxin in food are risk to liver cancer [44]. Studies included in this meta-analysis contained various cancers, ethnic populations and nations, and multifactor such as gender, age, lifestyle, culture barriers, access to health care and exposure to pathogens and carcinogens were disparate. While lacking of individual information inhibited us from controlling the possible confounding factors which might be caused by the inconsistencies above. We also could not perform more precise calculation of adjusted ORs and further analysis of potential gene-environment interactions. Secondly, included researches did not cover all kinds of digestive system cancers, such as pancreatic cancer. And thirdly, language bias might derive from the screened references of English documents only.

In summary, this meta-analysis indicated that miR-196a2 rs11614913 polymorphism may increase the susceptibility of digestive system cancers, especially of CRC and HCC. SNP in this locus may considerably act as a candidate of biomarker for cancer screening, diagnosis and therapy in the future. To confirm our findings, further well-designed studies with large sample size in diverse ethnic populations, more types of digestive system cancers along with tissue-specific biochemical, functional and expressional characteristics are required.

## Supporting Information

Figure S1 **Funnel plot of publication bias.** The standard error of log (OR) is plotted versus OR for each study. Each square represents a separate study for the indicated association by allele contrast (C vs. T). The dotted line in blue indicates the estimated OR.
(TIF)

Figure S2 **Forest plots of dominant model (CC/CT vs. TT) in different subgroups.** The squares and horizontal lines correspond to OR and 95% CI of specific study, and the area of squares reflects study weight (inverse of the variance). The diamond represents the pooled OR and its 95% CI.
(TIF)

Table S1 **Genotype frequency distribution of studies included.**
(DOC)

Table S2 **Scale for methodological quality assessment.**
(DOC)

Table S3 **Heterogeneity test.**
(DOC)

Table S4 **ORs (95% CI) of sensitivity analysis.**
(DOC)

Table S5 **MOOSE Checklist.**
(DOC)
