# Supplementary figures and images for: A Genetic Variant in miR-196a2 Increased Digestive System Cancer Risks: A Meta-Analysis of 15 Case-Control Studies
Source: PLoS One. 2012 Jan 24;7(1):e30585. doi: 10.1371/journal.pone.0030585 (PMC3265498; doi:10.1371/journal.pone.0030585)

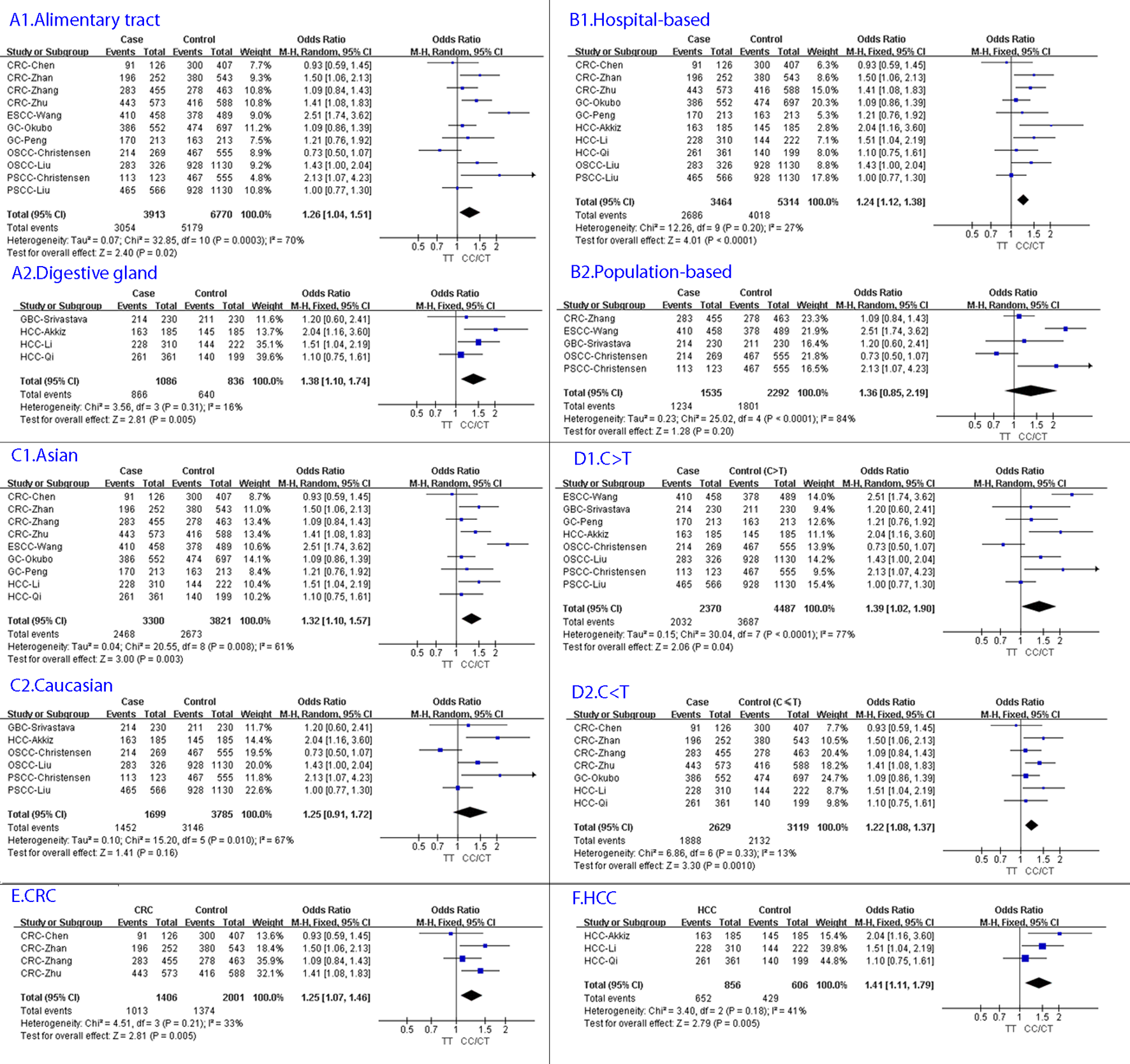

Supplement: Figure S2 — Forest plots of dominant model (CC/CT vs. TT) in different subgroups. The squares and horizontal lines correspond to OR and 95% CI of specific study, and the area of squares reflects study weight (inverse of the variance). The diamond represents the pooled OR and its 95% CI. (TIF) [file pone.0030585.s002.tif]
