# Supplementary material for: A Genetic Variant in miR-196a2 Increased Digestive System Cancer Risks: A Meta-Analysis of 15 Case-Control Studies
Source: PLoS One. 2012 Jan 24;7(1):e30585. doi: 10.1371/journal.pone.0030585 (PMC3265498; doi:10.1371/journal.pone.0030585)
Supplement: Table S1 — Genotype frequency distribution of studies included. (DOC) [file pone.0030585.s003.doc]

| **Table S1. Genotype frequency distribution of studies included.** | | | | | |
| --- | --- | --- | --- | --- | --- |
| First author | C frequency | Genotypes (Case/Control) | | | Cancer |
|  | in controls | TT | CT | CC | type |
| Zhan | 0.454 | 56/163 | 128/267 | 68/113 | CRC |
| Chen | 0.484 | 35/107 | 64/206 | 27/94 | CRC |
| Zhu | 0.457 | 130/172 | 303/295 | 140/121 | CRC |
| Zhang♫ | 0.388 | 172/185 | 204/197 | 79/81 | CRC |
| Wang | 0.517 | 48/111 | 262/250 | 148/128 | ESCC |
| Srivastava | 0.754 | 16/19 | 95/75 | 119/136 | GBC |
| Okubo | 0.429 | 166/223 | 281/350 | 105/124 | GC |
| Peng | 0.514 | 43/50 | 94/107 | 76/56 | GC |
| Li | 0.419 | 82/78 | 150/102 | 78/42 | HCC |
| Qi (HBV)♣ | 0.435 | 100/59 | 179/107 | 82/33 | HCC |
| Akkiz | 0.549 | 22/40 | 86/87 | 77/58 | HCC |
| Christensen♫ | 0.590 | 55/88 | 118/279 | 96/188 | OSCC |
| Liu♫ | 0.580 | 43/202 | 170/545 | 113/383 | OSCC |
| Christensen♫ | 0.590 | 10/88 | 63/279 | 50/188 | PSCC |
| Liu♫ | 0.580 | 101/202 | 303/545 | 162/383 | PSCC |
| ♫: unpublished data requested from corresponding researchers; ♣: HBV patients without HCC were controls and HBV patients with HCC were cases in the study of Qi. | | | | | |
