# Supplementary material for: A Genetic Variant in miR-196a2 Increased Digestive System Cancer Risks: A Meta-Analysis of 15 Case-Control Studies
Source: PLoS One. 2012 Jan 24;7(1):e30585. doi: 10.1371/journal.pone.0030585 (PMC3265498; doi:10.1371/journal.pone.0030585)
Supplement: Table S3 — Heterogeneity test. (DOC) [file pone.0030585.s005.doc]

| **Table S3. Heterogeneity test.** | | | | | |
| --- | --- | --- | --- | --- | --- |
| Stratification | CT vs. TT | CC vs. TT | CC/CT vs TT | CC vs CT/TT | C vs T |
|  | Ph, I² (%) | Ph, I² (%) | Ph, I² (%) | Ph, I² (%) | Ph, I² (%) |
| Digestive cancers | <0.01, 55 | <0.01, 65 | <0.01, 62 | 0.01, 52 | <0.01, 63 |
| Tumor site |  |  |  |  |  |
| Alimentary tract | <0.01, 64 | <0.01, 70 | <0.01, 70 | 0.04, 47 | <0.01, 65 |
| CRC | 0.47, 0 | 0.12, 49 | 0.21, 33 | 0.35, 8 | 0.15, 44 |
| GC | 0.85, 0 | 0.31, 4 | 0.70, 0 | 0.16, 49 | 0.23, 30 |
| ESCC | - | - | - | - | - |
| OSCC | <0.01, 87 | 0.07, 70 | 0.01, 85 | 0.82, 0 | 0.22, 32 |
| PSCC | 0.13, 55 | 0.01, 85 | 0.04, 75 | 0.02, 81 | 0.01, 84 |
| Digestive gland | 0.36, 7 | 0.34, 10 | 0.31, 16 | 0.02, 69 | 0.04, 65 |
| HCC | 0.22, 34 | 0.48, 0 | 0.18, 41 | 0.97, 0 | 0.42, 0 |
| GBC | - | - | - | - |  |
| Source of control |  |  |  |  |  |
| HB | 0.53, 0 | 0.02, 53 | 0.20, 27 | 0.01, 58 | <0.01, 59 |
| PB | <0.01, 83 | <0.01, 81 | <0.01, 84 | 0.10, 48 | <0.01, 75 |
| Ethnicity |  |  |  |  |  |
| Asian | 0.02, 55 | 0.02, 55 | <0.01, 61 | 0.52, 0 | 0.09, 41 |
| Caucasian | 0.02, 62 | <0.01, 70 | 0.01, 67 | 0.02, 64 | <0.01, 70 |
| Allele frequency in controls |  |  |  |  |  |
| C>T | <0.01, 78 | <0.01, 78 | <0.01, 77 | <0.01, 68 | <0.01, 76 |
| C≤T | 0.59, 0 | 0.23, 26 | 0.33, 13 | 0.50, 0 | 0.24, 25 |
| Ph: P-value of Q-test for heterogeneity identification; I² index: a quantitative measurement which indicates the proportion of total variation in study estimates that is due to between-study heterogeneity. | | | | | |
