# Supplementary material for: A Genetic Variant in miR-196a2 Increased Digestive System Cancer Risks: A Meta-Analysis of 15 Case-Control Studies
Source: PLoS One. 2012 Jan 24;7(1):e30585. doi: 10.1371/journal.pone.0030585 (PMC3265498; doi:10.1371/journal.pone.0030585)
Supplement: Table S4 — ORs (95% CI) of sensitivity analysis. (DOC) [file pone.0030585.s006.doc]

| **Table S4**. **ORs (95% CI) of sensitivity analysis.** | | | | | |
| --- | --- | --- | --- | --- | --- |
| Excluding literature | CT *vs.* TT | CC *vs.* TT | Dominant model | Recessive model | C *vs.* T |
| one by one | OR (95% CI) P*h* | OR (95% CI) P*h* | OR (95% CI) P*h* | OR (95% CI) P*h* | OR (95% CI) P*h* |
| Over all | 1.25(1.07-1.45)0.005 | 1.38(1.13-1.67)0.0003 | 1.29(1.10-1.50)0.0007 | 1.14(1.01-1.30)0.01 | 1.15(1.05-1.26)0.0005 |
| Zhan 2011 | 1.24(1.06-1.46)0.003 | 1.35(1.10-1.66)0.0003 | 1.27(1.08-1.50)0.0006 | 1.13(0.99-1.28)0.01 | 1.13(1.03-1.25)0.0007 |
| Chen 2010 | 1.27(1.09-1.49)0.004 | 1.41(1.16-1.73)0.0003 | 1.31(1.12-1.54)0.0007 | 1.16(1.02-1.32)0.008 | 1.16(1.06-1.27)0.0005 |
| Zhu 2011 | 1.24(1.05-1.46)0.004 | 1.37(1.11-1.69)0.0002 | 1.28(1.08-1.51)0.0005 | 1.14(0.99-1.30)0.009 | 1.14(1.03-1.26)0.0005 |
| Zhang 2011 | 1.27(1.07-1.49)0.003 | 1.41(1.15-1.74)0.0003 | 1.31(1.10-1.55)0.0005 | 1.16(1.01-1.32)0.008 | 1.16(1.05-1.27)0.0004 |
| Wang 2010 | **1.18(1.07-1.30)0.15** | 1.30(1.09-1.54)0.01 | 1.21(1.06-1.38)0.05 | 1.13(0.99-1.29)0.01 | 1.12(1.03-1.23)0.005 |
| Srivastava 2010 | 1.24(1.06-1.45)0.003 | 1.40(1.14-1.71)0.0002 | 1.29(1.10-1.51)0.0004 | 1.17(1.04-1.33)0.03 | 1.16(1.06-1.27)0.001 |
| Okubo 2010 | 1.27(1.08-1.50)0.004 | 1.40(1.14-1.73)0.0002 | 1.31(1.10-1.55)0.0006 | 1.15(1.01-1.32)0.007 | 1.15(1.05-1.27)0.0004 |
| Peng 2010 | 1.27(1.08-1.48)0.004 | 1.37(1.11-1.68)0.0002 | 1.29(1.10-1.52)0.0004 | 1.12(0.99-1.28)0.02 | 1.14(1.04-1.25)0.0004 |
| Li 2010 | 1.24(1.06-1.46)0.003 | 1.35(1.11-1.68)0.0003 | 1.27(1.08-1.50)0.0005 | 1.13(0.99-1.29)0.01 | 1.13(1.03-1.25)0.0006 |
| Qi (HBV) 2010 | 1.27(1.09-1.49)0.004 | 1.37(1.12-1.69)0.0002 | 1.30(1.10-1.54)0.0004 | 1.13(0.99-1.28)0.01 | 1.15(1.04-1.26)0.0003 |
| Akkiz 2011 | 1.23(1.05-1.43)0.005 | 1.34(1.10-1.62)0.0006 | 1.26(1.08-1.47)0.001 | 1.12(0.99-1.28)0.02 | 1.13(1.03-1.24)0.001 |
| Christensen(OSCC) 2010 | 1.30(1.13-1.49)0.05 | 1.43(1.18-1.74)0.001 | 1.33(1.15-1.55)0.006 | 1.15(1.01-1.32)0.007 | 1.16(1.06-1.28)0.0009 |
| Liu 2010(OSCC) 2010 | 1.24(1.05-1.45)0.004 | 1.38(1.12-1.70)0.0002 | 1.28(1.08-1.51)0.0005 | 1.16(1.01-1.33)0.007 | 1.15(1.04-1.27)0.0003 |
| Christensen(PSCC) 2010 | 1.23(1.06-1.43)0.005 | 1.34(1.10-1.64)0.0004 | 1.26(1.08-1.47)0.0009 | 1.13(1.00-1.29)0.009 | 1.14(1.04-1.25)0.0005 |
| Liu 2010(PSCC) 2010 | 1.27(1.07-1.49)0.004 | 1.44(1.19-1.74)0.004 | 1.32(1.12-1.55)0.001 | **1.19(1.07-1.32)0.18** | 1.17(1.08-1.28)0.01 |
| Ph: P-value of Q-test for heterogeneity test; OSCC: oral cavity squamous cancer; PSCC: pharynx squamous cancer; The results marked in boldface represent crucial influence to overall pooled ORs. | | | | | |
