# Supplementary material for: A Genetic Variant in miR-196a2 Increased Digestive System Cancer Risks: A Meta-Analysis of 15 Case-Control Studies
Source: PLoS One. 2012 Jan 24;7(1):e30585. doi: 10.1371/journal.pone.0030585 (PMC3265498; doi:10.1371/journal.pone.0030585)
Supplement: Table S5 — MOOSE Checklist. (DOC) [file pone.0030585.s007.doc]

**MOOSE Checklist**

| **Criteria** | | **Brief description of how the criteria were handled in the meta-analysis** |
| --- | --- | --- |
| **Reporting of background should include** | |  |
| √ | Problem definition | miRNAs play crucial roles in the various biological processes including cell growth regulation, differentiation, apoptosis and tumorigenesis. The association between miR-196a2 polymorphism and the digestive cancer risks were inconsistent in previous studies. |
| √ | Hypothesis statement | miR-196a2 polymorphism might influence the susceptibility of digestive cancers, and which might relate to the ethnicity, cancer type or other aspects. |
| √ | Description of study outcomes | Digestive system cancer |
| √ | Type of exposure or intervention used | CT, CC, CT/CC genotypes or C allele in miR-196a2 |
| √ | Type of study designs used | We included case-control studies. |
| √ | Study population | We placed no restriction. |
| **Reporting of search strategy should include** | |  |
| √ | Qualifications of searchers | The credentials of all investigators are indicated in the author list. |
| √ | Search strategy, including time period included in the synthesis and keywords | Electronic database: PubMed 1965 – 20^th^ August 2011  Retrieving query formulation: (microrna 196a2 OR rs11614913) polymorphisms cancer. |
| √ | Databases and registries searched | PubMed. |
| √ | Search software used, name and version, including special features | We did not employ any search software. EndNote was used to merge retrieved citations and eliminate duplications. |
|  | Use of hand searching | We did not browse any relative bibliographies by hand. |
| √ | List of citations located and those excluded, including justifications | Details of the literature search process are outlined in the flow chart. The citation list is available upon request. We did not show the exclusion list. |
|  | Method of addressing articles published in languages other than English | We just searched the documents in English and paid no more attentions to other languages. |
| √ | Method of handling abstracts and unpublished studies | We obtained 5 unpublished data sets in which one data set was provided by author MZ of this article, and the other 4 were requested from two scientists who published relative studies while did not showed the data we needed in their articles. |
| √ | Description of any contact with authors | We contacted with researchers who had conducted relative studies while did not reported the genotype frequency we needed in their published articles. |
| **Reporting of methods should include** | |  |
| √ | Description of relevance or appropriateness of studies assembled for assessing the hypothesis to be tested | Detailed inclusion and exclusion criteria were described in the methods section. |
| √ | Rationale for the selection and coding of data | Data extracted from each of the studies were relevant to the population characteristics, study design, exposure, outcome, and possible effect modifiers of the association. |
| √ | Assessment of confounding | Subgroup analysis was performed and we conducted sensitivity analysis by deleting a single study one by one for each time. |
| √ | Assessment of study quality, including blinding of quality assessors; stratification or regression on possible predictors of study results | We assessed the methodological qualities of included studies by scoring according to a designed scale. Each study obtained an acceptable score(≧6). The results of sensitivity analyses were relatively stable. |
| √ | Assessment of heterogeneity | Cochrane’s Q test and the I²index were performed to explore the heterogeneity. |
| √ | Description of statistical methods in sufficient detail to be replicated | Methods of heterogeneity test, quantitative synthesis, assessments of publication bias, sensitivity analyses are detailed in the methods. |
| √ | Provision of appropriate tables and graphics | We provided 1 figure of flow chart to explain the article searching; 1 table for study characteristics; 1 table for pooled analysis; 1 figure of funnel plot for the examination of publication bias;1 table for genotype distribution; 1 table for study’ quality assessment; 1 table for heterogeneity test; 1 table for sensitivity analysis. |
| **Reporting of results should include** | |  |
|  | Graph summarizing individual study estimates and overall estimate | We did not provide forest graph for pooled analysis. |
| √ | Table giving descriptive information for each study included | Table 1 and Table S1. |
| √ | Results of sensitivity testing | Table S4. |
| √ | Indication of statistical uncertainty of findings | 95% confidence intervals were presented with all summary estimates, *P* values and results of sensitivity analyses |
| **Reporting of discussion should include** | |  |
| √ | Quantitative assessment of bias | Sensitivity analyses indicate this non-significant association was stable. |
| √ | Justification for exclusion | Reviewers or meta-analysis, studies on non-digestive cancer and other irrelevant studies which were inconsistent to the inclusion criteria were excluded. |
|  | Assessment of quality of included studies | We did not discuss the results of the sensitivity analyses. |
| **Reporting of conclusions should include** | |  |
| √ | Consideration of alternative explanations for observed results | We discussed that potential unmeasured confounders such as differences in various cancer susceptibilities of various tissues, life style, environmental factor and relative small sample size might cause different results. |
| √ | Generalization of the conclusions | We found significant association between miR-196a2 polymorphism and increased susceptibility of digestive system cancers, especially of CRC, HCC and Asians. Besides, C allele may contribute to increased digestive cancer risks. |
| √ | Guidelines for future research | Further well-designed studies with large sample size in diverse ethnic populations, more types of digestive system cancers along with tissue-specific biochemical, functional and expressional characteristics are required. |
| √ | Disclosure of funding source | This study was supported by the National Natural Science Foundation of China (No. 81072356). |
